# Sequence characteristics define trade-offs between on-target and genome-wide off-target hybridization of oligoprobes

**DOI:** 10.1371/journal.pone.0199162

**Published:** 2018-06-21

**Authors:** Olga V. Matveeva, Aleksey Y. Ogurtsov, Nafisa N. Nazipova, Svetlana A. Shabalina

**Affiliations:** 1 Biopolymer Design LLC, Acton, Massachusetts, United States of America; 2 National Center for Biotechnology Information, National Library of Medicine, National Institutes of Health, Bethesda, Maryland, United States of America; 3 Institute of Mathematical Problems of Biology, RAS – the Branch of Keldysh Institute of Applied Mathematics of Russian Academy of Sciences, Pushchino, Moscow Region, Russia; University of Helsinki, FINLAND

## Abstract

Off-target oligoprobe’s interaction with partially complementary nucleotide sequences represents a problem for many bio-techniques. The goal of the study was to identify oligoprobe sequence characteristics that control the ratio between on-target and off-target hybridization. To understand the complex interplay between specific and genome-wide off-target (cross-hybridization) signals, we analyzed a database derived from genomic comparison hybridization experiments performed with an Affymetrix tiling array. The database included two types of probes with signals derived from (i) a combination of specific signal and cross-hybridization and (ii) genomic cross-hybridization only. All probes from the database were grouped into bins according to their sequence characteristics, where both hybridization signals were averaged separately. For selection of specific probes, we analyzed the following sequence characteristics: vulnerability to self-folding, nucleotide composition bias, numbers of G nucleotides and GGG-blocks, and occurrence of probe’s *k*-mers in the human genome. Increases in bin ranges for these characteristics are simultaneously accompanied by a decrease in hybridization specificity—the ratio between specific and cross-hybridization signals. However, both averaged hybridization signals exhibit growing trends along with an increase of probes’ binding energy, where the hybridization specific signal increases significantly faster in comparison to the cross-hybridization. The same trend is evident for the *S* function, which serves as a combined evaluation of probe binding energy and occurrence of probe’s *k*-mers in the genome. Application of *S* allows extracting a larger number of specific probes, as compared to using only binding energy. Thus, we showed that high values of specific and cross-hybridization signals are not mutually exclusive for probes with high values of binding energy and *S*. In this study, the application of a new set of sequence characteristics allows detection of probes that are highly specific to their targets for array design and other bio-techniques that require selection of specific probes.

## Introduction

Many biotechnology applications involve oligoprobe hybridization with complementary targets in DNA or RNA as a basic procedural step. One such application is microarray technology. High throughput sequencing is gradually replacing microarrays as the preferred method for studying cellular transcript expression levels. However, microarrays are still dominating certain applications, such as identification of transcription binding sites [1], and gene copy number evaluations and genotyping [2–3]. It is possible to envision a powerful symbiosis between microarrays and new generation sequencing technologies [4].

Desirable reactions between an oligoprobe and its complementary target are frequently complicated by other undesirable interactions of the probe. Particularly problematic is off-target probe binding with partially complementary DNA or RNA sequences, which are almost always present in a hybridization mixture. These interactions happen in parallel with on-target interactions. Microarray hybridization is an excellent technology for characterizing an oligoprobe’s on-target and off-target interactions. A single microarray experiment, especially with comparative genomic hybridization (CGH) [5–6], allows visualization of millions of hybridization reactions. Despite many artifacts [7], no other technology provides such a high volume of useful information for analysis of specific oligoprobe-target interaction in a complex mixture of nonspecific reactions. A microarray hybridization signal consists of two components, target-specific and cross-hybridization. The ratio of these two components is a measure of hybridization specificity. The most specific probes produce the most reliable results.

One microarray application is the evaluation of gene copy number variation, and can be performed using CGH experiments [8] with Affymetrix tiling arrays that cover a whole genome with 25nt probes. Variation among individual probe signals is a huge drawback of array technology in general and, in particular, Affymetrix arrays. During post-experimental data analysis, signals from different tiled probes are averaged using a sliding window. Such averaging helps diminish the signal variability problem and allows better detection of deleted gene regions and those with variable copy numbers. However, signal averaging alone cannot eliminate the variability problem.

Hybridization signals derived from one microarray experiment even with the same target concentration are variable. Uneven hybridization conditions [9], target RNA quality [10–11], probe’s vulnerability to non-Watson Crick (non-WC) interactions through G-blocks [12–18], probe’s secondary structure [19–21] and probe’s synthesis failure [22], all play a role in signal variability. Hybridization signals are also affected by the probe’s binding energy, which defines the probe’s ability to form stable oligo-target duplexes [23–26]. Finally, different cross-hybridization signal components contribute to the signal’s variability.

A number of studies have analyzed factors that influence genome-wide cross-hybridization levels of microarray probes. Duplexes of 10–16 nucleotides that are complementary to targets may be sufficient to generate a cross-hybridization signal [27–28]. For 50-nt probes in particular, it was noted that “a complementary stretch of nucleotides as short as 12 nucleotides may result in the appearance of significant signal from an unintended binding partner” [28]. Shorter probes (25- nt) are hybridized in less stringent conditions compared to longer probes (50-nt). Therefore, much shorter complementary stretches might significantly contribute to the cross-hybridization signal. Kapur and co-authors [29] proposed a filtering method to detect and remove probes that have certain sequence-specific alignments with off-target transcripts. Similar approaches for filtering out potentially non-specific probes were suggested by others [30–31]. In these studies, the authors calculate a probe’s “uniqueness score” by evaluating the probe’s substrings frequency occurrence in a targeted genome. The main flaw of these studies [29–31] is a lack of consideration for the probe’s binding energies, which were shown to correlate significantly with cross-hybridization intensity [32–33]. Very few existing cross-hybridization models consider not only probe sequence similarity with non-target sequences, but also its thermodynamic features, including its binding energy [34–35].

To standardize terminology definition, we recommend that the scientific community discriminate between absolute cross-hybridization signals and relative cross-hybridization values. The term “absolute” cross-hybridization is used to identify signals that derive from probes that interact with partially complemented (off target) sequences only, e.g. without fully complemented targets. The term “relative” cross-hybridization represents the proportion of absolute cross-hybridization in an overall hybridization signal. Finally, the overall hybridization signal is represented by a sum of target specific and absolute cross-hybridization signals.

Why would such terminology and discrimination be useful for microarray hybridization studies? Two probes might have similar absolute, but different relative, cross-hybridization values. The latter is more important for probe design than the former. Relative cross-hybridization in an optimal probe should be low, whereas the same is not necessary for absolute cross-hybridization. Moreover, probes with low absolute cross-hybridization might have a low specific signal component and might be unsuitable for sensitive target detection and, consequently, for array design. A limited number of studies have analyzed relationships between probes’ sequence characteristics and target specific and/or cross-hybridization signals: two publications describe such analysis for 50-nt [36] and 25-nt [37] oligoprobes, respectively.

This study describes the analysis of relationships between sequence characteristics and hybridization signals of probes. The focus of this study is not limited to hybridization specificity or cross-hybridization signals. We have concentrated on the difference between absolute and relative cross-hybridizations and their divergent behavior according to changes in various sequence characteristics. These findings could be used for further optimization of recent advanced probe-target hybridization models [35] as well as for improvement of probes’ design.

## Materials and methods

### Hybridization database

**Fig 1:**
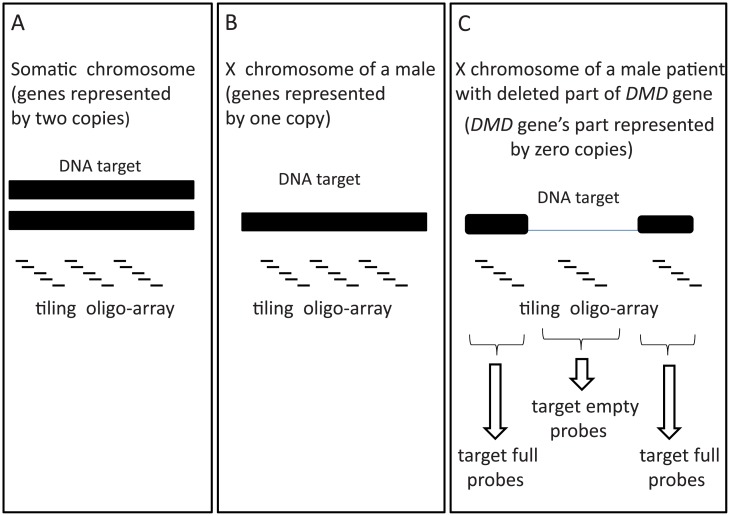
Hybridization experiment scheme with tiling microarray. The DNA target region is represented by (A) two gene copies, (B) one copy or (C) no gene—one copy (X chromosome) combined with deletion of DMD gene fragment in a male patient.

In normal human somatic chromosomes, each gene is represented by two copies (Fig 1A). In the male X chromosome, most genes are represented by one copy (Fig 1B). In male patients affected by Duchenne muscular dystrophy (DMD), a region of the DMD gene is deleted and consequently represented by zero copies (Fig 1C).

We analyzed hybridization data from an experiment performed with DNA from a DMD-affected male patient. A large part of the DMD gene in the X chromosome was deleted from the patient’s DNA. Consequently, the oligoprobes targeting the deleted region of the DMD gene were without specific targets and produced only genomic cross-hybridization signals. Oligoprobes that targeted the non-deleted region in the X chromosome, where a specific target is present, represented the sum of target-specific and cross-hybridization signals. This sum is referred to as the “overall hybridization” in this study. Each set of probes with and without targets included 10^4^ data points from the same hybridization experiment, performed with the same chip (Fig 1C). The probe dataset was provided courtesy of the Department of Human Genetics, University of Utah (the patient provided written consent for using his bio-samples for genomic and genetic research; data are available by requests). The standard Affymetrix protocol was used for genomic DNA amplification and hybridization at 45°C. Hybridization was performed using a tiling array Gene Chip Human Mapping 100K Set.

### Definitions

**Fig 2:**
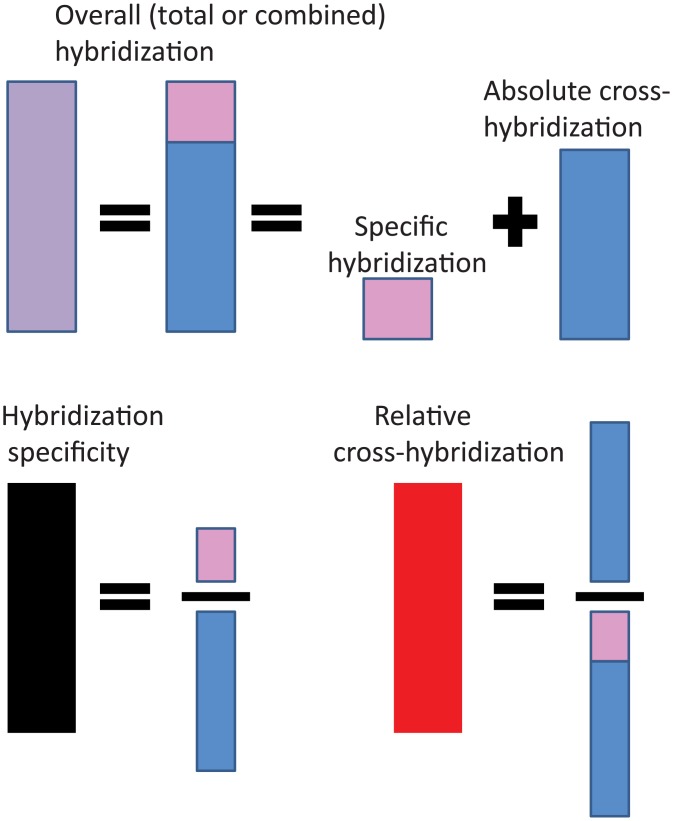
Schematic representation of probes’ hybridization characteristics.

The main hybridization probes’ characteristics of the study are illustrated graphically in Fig 2. Based on previous studies [37] we assume that for sets of probes with very similar sequence characteristics averaged genome-wide cross-hybridization signals are also similar, regardless of whether these probes were with or without targets.

Overall hybridization (O) is represented by signals from probes with targets. Overall hybridization combines target specific and cross-hybridization signals.

Absolute cross-hybridization (A) is represented by signals that derive from interactions of probes with partially complemented (off target) sequences only, e.g. without fully complemented targets.

Relative cross-hybridization (R) is the proportion of absolute cross-hybridization in the overall hybridization signal: *R = A/O*

Target Specific hybridization (Sh) is the difference between overall hybridization and absolute cross-hybridization: *Sh = O* − *A*

Hybridization specificity (HS) is the ratio between target-specific and absolute cross-hybridizations: *HS* = *Sh/A* = (*O-A*)/*A* = *1/R-1*

### Sequence characteristics of probes

#### Genomic occurrence of *k*-mers

We downloaded publicly available human genome sequences for the GRCh38.p7 version of the genome assembly ftp://ncbi.nlm.nih.gov/genomes/H_sapiens/ and created a table of occurrences of *k*-mers (where 7 ≤ *k* ≤ 11) for the human chromosomes. The frequencies of *k*-mers (where 7 ≤ *k* ≤ 11) we define as a number of occurrences of the *k*-mer normalized by total number of all *k*-mers occurrences in the human chromosomes. For each oligonucleotide probe in our set of 20K oligonucleotides, we calculated the minimum, maximum and total number of occurrence of all *k*-mers: fifteen 11-mers, sixteen 10-mers, seventeen 9-mers, eighteen 8-mers and nineteen 7-mers, presented in the 25-nt oligonucleotide passenger strand. Genomic occurrence of each *k*-mer in an oligoprobe was calculated using in-house scripts [38–40]. In this study, “genomic occurrence of 11-mers” was assigned to each probe as a minimum among all 11-mers in a probe that reflects the accessibility of the most unique seed region of the probe.

#### Estimation of *S* function, theoretical hybridization specificity

We estimate theoretical values of *S* function using the earlier described model and approach for calculation of predicted probes’ specificity [37]. Calculation of theoretical hybridization specificity *S* is based on the following formulas:

$$S_{olig}=\frac{e^{-\frac{{\Delta G}_{spec}}{RT}}}{X_{olig}},\;\text{where}\;X_{olig}=\sum_{i}e^{-\frac{{\Delta G}_{cross,i}}{RT}}$$

where for each oligo, function *S* is the ratio between predicted specific signal (numerator) and predicted accumulative cross-hybridization signal (denominator); *ΔG*_*spe*c_ is the free energy change related to the reaction of fully paired duplex formation between an oligoprobe and target sequence; *ΔG*_*cross*_ is the free energy change related to the reaction of duplex formation between an oligoprobe and partially complementary sequence in genomic DNA; *X*_*olig*_ is the theoretical estimation of the accumulative cross-hybridization component of the oligonucleotide. Assuming every target has a core of 7-11nt of exact complementarity, we can estimate *X*_*olig*_ by the following expression:

$$X_{olig}=\underset{n,i}{\text{max}}\left({N_{n,i}*e}^{-\frac{{\Delta G}_{n,i}}{RT}}\right),\;\text{where}\;n=7..11,i=1..(25-n+1)$$

where *i* is a position of *n*-mer in the oligonucleotide, and *N*_*n*,*i*_ − the total number of occurrences of *n*-mer (started from *i* position of *n*-mer in the oligonucleotide) in the human genome.

#### Calculation of nucleotide composition bias for oligoprobes

We have recently evaluated the sequence complexity and sequence asymmetry (SC and SA) scores [37] based on the nucleotide occurrence in a probe. In this study, a similar approach is used for the estimation of oligoprobe nucleotide composition bias using sequence asymmetry and simplicity score (SAS).

The sum of the squared differences between frequencies of A and T nucleotides and of G and C nucleotides represents the SAS score of a probe: *SAS* = (*f*_*A*_ − *f*_*T*_)^2^ + (*f*_*G*_ − *f*_*C*_)^2^, where *SAS* is the sequence asymmetry and simplicity score of a probe, and *f*_*N*_ is a frequency of *N* nucleotide (*N* = *A*, *T*, *G* or *C*) in a probe.

Theoretically, the SAS score’s range varies from 0 to 1, where 0 corresponds to probes with equal proportions of each nucleotide and 1 corresponds to a probe that consists of only a single type of nucleotide. So, for example a score of 1 would represent a probe comprised entirely of As. In this database, the SAS score values range from 0.0015 to 0.4.

#### Oligo probe self-folding potential (secondary structure) and binding energy (probe-target duplex stability)

The probe’s self-folding and binding energies were evaluated by calculating *ΔG*_folding_ and *ΔG*_binding_ respectively. *ΔG*_folding_ was calculated by the A-Fold software [41–42], while *ΔG*_binding_ was evaluated using in-house scripts and previously published nearest neighbor parameters [43–45].

#### Binning and averaging procedure

Averaging of hybridization signals from consecutively positioned tiled probes targeting the same gene is a routine procedure for CGH data analysis. Such averaging diminishes problems related to signal variability from individual probes, and in turn improves detection of deleted gene regions versus those with variable copy numbers. We applied a signal averaging procedure to analyzed data, where we sorted probes into bins according to their sequence characteristics and then averaged signals from each bin. The list of probe characteristics that was used as categorization criteria for binning and averaging included G-count, GGG-block count, nucleotide composition bias measured as SAS score (see below), self-folding, genomic occurrence of 11-mers, binding energy (*ΔG*) and theoretically estimated function *S*, where *S* is calculated based on the combined evaluations of probe binding energy and genomic occurrence of *k*-mers (see above).

## Results

In this study, we discriminate between absolute and relative cross-hybridization values using two types of probes with signals derived from (i) a combination of specific signal and cross-hybridization or (ii) genomic cross-hybridization only. Absolute cross-hybridization applies to cross-hybridization signal that derives from contributions of all off-target interactions of each probe. The relative cross-hybridization term applies to a proportion of absolute cross-hybridization in an overall probe’s signal, which includes two components: the target specific signal and cross-hybridization. Therefore, while the absolute cross-hybridization represents a signal, the relative one represents a calculated signals’ ratio.

All probes from the database were categorized into bins according to their sequence characteristics and hybridization signals in each bin were averaged. The difference between bins with averaged signals of probes with and without a target represents averaged specific hybridization for all probes characterized by similar sequence characteristic. The calculation of the difference was used for evaluation of hybridization specificity, which is a ratio between specific- and absolute cross-hybridizations. The binning and averaging approach allowed finding and analyzing trends in relationships between probes’ hybridization and sequence characteristics without knowledge of all hybridization characteristics of each individual probe.

### Sequence characteristics of probes that affect hybridization signals

#### G-effects

**Fig 3:**
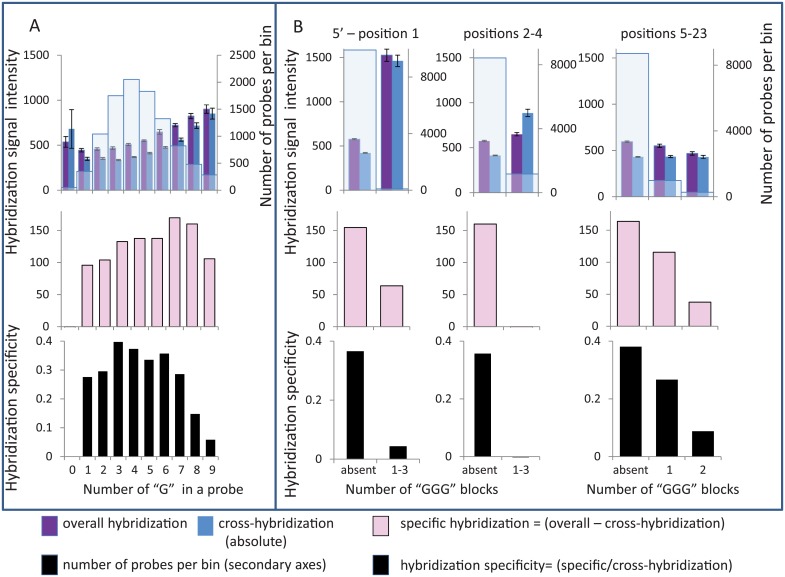
G-effects influence on hybridization specificity. Categorization of oligoprobes according to numbers of G nucleotides (A) or GGG-blocks (B) is presented. Averaged hybridization signals were calculated for each bin and their values are shown along primary Y axes as columns of assorted colors. Overall (purple) hybridization and cross-hybridization (dark blue) are shown on the upper panels; specific hybridization (pink) is presented on the middle panels. Hybridization specificity (black), defined as ratio between specific and cross-hybridization, is presented on the lower panels. Numbers of the probes in each bin are shown along the secondary Y axes as light blue columns on the upper panels. A. Relationship between probes’ hybridization signals and the numbers of G nucleotides. B. Relationship between probes’ hybridization signals and the numbers of GGG-blocks in the positions of the probe. Probes were categorized into bins according to GGG-block counts in the probe’s positions from 1 to 23 (numeration from probe’s 5’ end). Location of GGG-blocks was assigned for three groups: for position 1, positions from 2^nd^ to 4^th^, and positions from 5 to 23^rd^. The left histograms show averaged signals for probes with GGG-blocks located at the first position of the probe, middle histograms show results for probes with GGG-blocks located in the 2^nd^ to 4^th^ positions. The numbers of GGG-blocks in any position from the 5^th^ to 23^rd^ is presented on the right histogram.

Hybridization specificity is negatively affected by high G-count and/or by G-block presence in a probe [37]. We collectively refer to this negative dependence as “G-effects”. The effects become stronger when G-count or G-block numbers increase in the probe. G-effects are responsible for high absolute (Fig 3) and high relative cross-hybridizations (Figure A-D in S1 Fig). G-count in probes varies from 0 to 15. Number of GGG blocks varies from 0 to 5. The sets of probes with G-count above 7nt (Fig 3A) and with more than one G-block (Fig 3B and 3C) have the lowest hybridization specificity. The ratios between specific and cross-hybridizations differ 10 times between probes with two GGG-blocks and without GGG-blocks.

**Fig 4:**
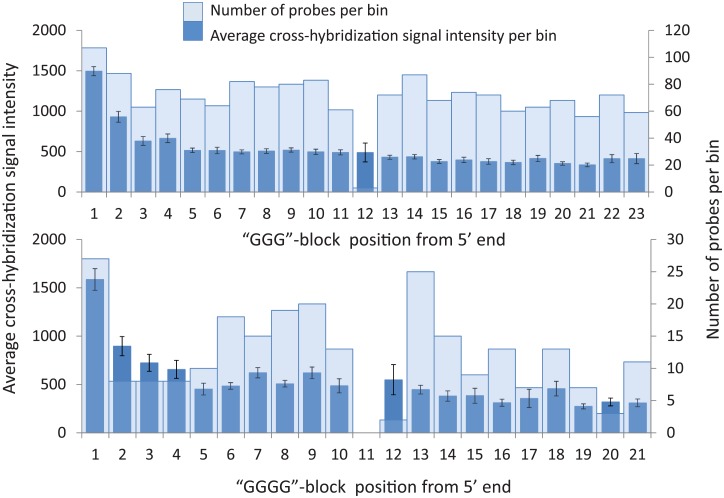
Averaged cross-hybridization signals of probes with various locations of GGG(G)-blocks. The upper panel shows results of probes’ binning and averaging for GGG-blocks and the lower panel for GGGG-blocks along the probe positions from the 1^st^ to 23^rd^ (5’→ 3’). Averaged absolute cross-hybridization signals are shown along the primary Y axes as dark blue columns and the numbers of probes in each bin are shown along the secondary Y axes as light blue columns.

Multiple studies suggest that array probes with four G nucleotides in a row (GGGG-block) are responsible for hybridization artifacts because of their involvement in non-WC interactions [12–18]. We found that probes with three Gs in a row behave almost as poorly as those with four (compare Fig 4 top and bottom histograms). These probes have high absolute cross-hybridization, especially if a G-block is located at probes’ 5’ end, which gradually diminishes as the position of the G-block moves from the 5’ end toward the 3’ end (Fig 4). The probes with one or more GGG blocks have very low hybridization specificity and, consequently, very high absolute and relative cross-hybridizations (Fig 3B and 3C).

#### SAS score

**Fig 5:**
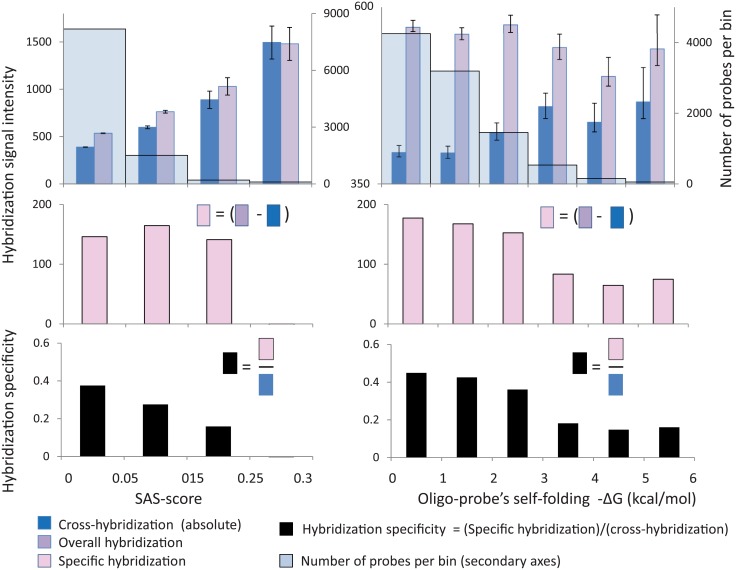
Nucleotide bias and folding potential affect hybridization specificity. Oligoprobes were categorized into bins according to nucleotide bias (SAS-score, left panel) and self-folding vulnerability (right panel). Averaged hybridization signals are shown along the primary Y axis and numbers of probes in each bin are shown along secondary Y axes as light blue columns on the top histograms. Averaged values of overall (dark blue) and cross-hybridization (grey) signals are shown on the top histograms, specific hybridization (pink) is shown on the middle histograms and hybridization specificity (black) is present on the bottom histograms. Numbers of probes in each bin are shown along the secondary Y axis as light blue columns on the top histograms.

We measured each probe’s nucleotide bias by calculating the probe’s SAS score (see Materials and methods), which varies from 0.0015 to 0.4 correspondingly. SAS score correlates negatively with hybridization specificity and positively with absolute (Fig 5, left panel) and with relative cross-hybridization (Figure E in S1 Fig).

#### Self-folding

Open probes with low self-folding potential are more specific with low absolute (Fig 5, right panel) and relative cross-hybridizations (Figure F in S1 Fig). *ΔG* values of probe’s self-folding varies from -14 to 0 kcal/mol. The hybridization specificity of comparatively open probes (-*ΔG* ≤ 2 kcal/mol) is at least twice as high versus the specificity of those with high self-folding vulnerability (-*ΔG* > 3 kcal/mol).

#### Genomic occurrence of *k*-mers

**Fig 6:**
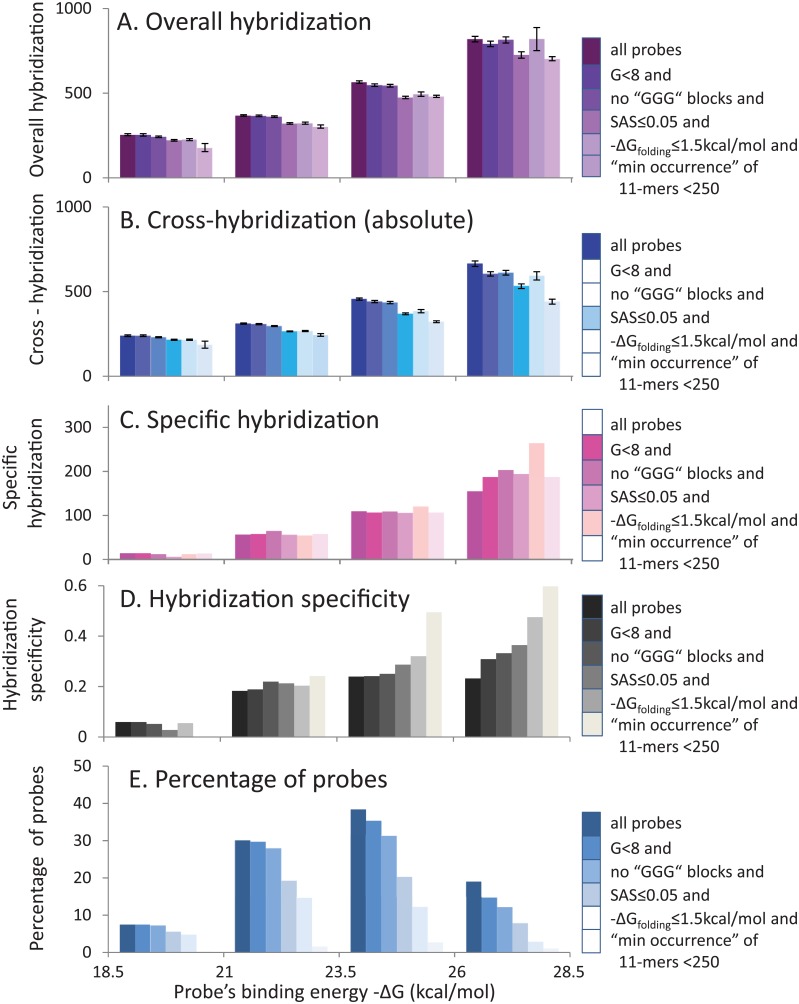
Categorization of probes by binding energy after filtration using all analyzed parameters. Oligoprobes were categorized into bins according to their binding energy. Averaged hybridization signals and other hybridization characteristics calculated for each bin are shown as columns of assorted colors. The columns with variable shades of a particular color illustrate the filtration process that gradually removes from the database all probes with a defined sequence characteristic, which negatively affect hybridization specificity because of involvement in parallel hybridization reactions. Colors of the columns becomes lighter after each filtration step. The darkest columns represent all probes from the database, while lightest columns represent probes after all filtration steps. The filtration process entails the following sequential probe removal steps: 8 or more of G in a sequence, at least one GGG block, SAS score above 0.05, -*ΔG* folding above 1.5 kcal/mol and minimum genomic occurrence among all 11-mers in oligo above 250. A. Overall hybridization (derived from the dataset of probes with specific targets). B. Cross-hybridization (absolute) (derived from the dataset of probes without specific targets). C. Specific hybridization (represented by subtraction values between overall and cross-hybridizations). D. Hybridization specificity (represented by ratio between specific and cross-hybridization). E. The percentage of probes (from complete dataset) in each bin.

We found that measurement of genomic occurrence of 11-mers in oligoprobes can be used for further increasing hybridization specificity. The minimum values of genomic occurrence of all 11-mers in a probe are particularly suitable for this purpose. Filtering out all probes with minimum values above 250 caused a decrease of cross-hybridization and an increase of hybridization specificity (Fig 6D).

#### Binding energy

Probes’ binding energy *ΔG* values vary from -16 to -33 kcal/mol. Probes’ overall hybridization and their absolute cross-hybridization values increase with probe binding energy (Fig 6A and 6B). Probes’ specific hybridization (Fig 6C) and hybridization specificity (Fig 6D) demonstrates a growing trend. In contrast, relative cross-hybridization has a descending trend (Figure G in S1 Fig). The specificity of probes with a particularly high binding energy (26 ≤ -*ΔG* ≤ 28.5 kcal/mol) is at least three times higher versus the specificity of probes with low binding energy (18 ≤ -*ΔG* ≤ 21.5 kcal/mol). Moreover, hybridization specificity of probes with the lowest binding energy (-*ΔG* < 18 kcal/mol) is close to zero (Fig 6D). Approximately 70% of probes in the database have -*ΔG* < 26 kcal/mol (Fig 6E).

All sequence characteristics described above, except binding energy, were used for filtering of oligoprobes with low hybridization specificity. After each filtering step, we reanalyzed hybridization signals of remaining probes, separated into bins according to their binding energy. Hybridization specificity of the remaining probes increased after extraction of probes with low specificity due to G-effects, high nucleotide bias or sequence asymmetry (SAS-score), and high self-folding potential. Each step of probe removal improved specificity of remaining probes, independent from the order of the applied filtration parameters. The effect that increased specificity was more pronounced among the probes with high binding energy. The specificity of probes with maximal binding energy (26 ≤ -*ΔG* ≤ 28.5 kcal/mol) more than doubled after filtration steps described above were applied (Fig 6D).

The removal of probes involved in parallel hybridization reactions, before probe categorization according to binding energy, unmasks trends in behavior of the remaining probes. Among these remaining probes the relationship between binding energy and hybridization specificity is stronger (Fig 6D).

#### Estimation of the *S* function

Based on *ΔG* and genome occurrence of *k*-mers included in the oligoprobes, the theoretical prediction of cross-hybridization, *X*_*olig*_, was estimated using a modification of recently published model (see Material & methods, [37]). The estimated values significantly correlated to experimental cross hybridization signals (*R* = 0.6, *P* < 3*10^−10^) and could be used for the prediction of model experiments. We also estimated the function *S*, related to the predicted probe’s specificity, using the earlier described approach [37], where *X*_*olig*_, the theoretical estimation of accumulative cross-hybridization, was used as the denominator (see Material & methods).

**Fig 7:**
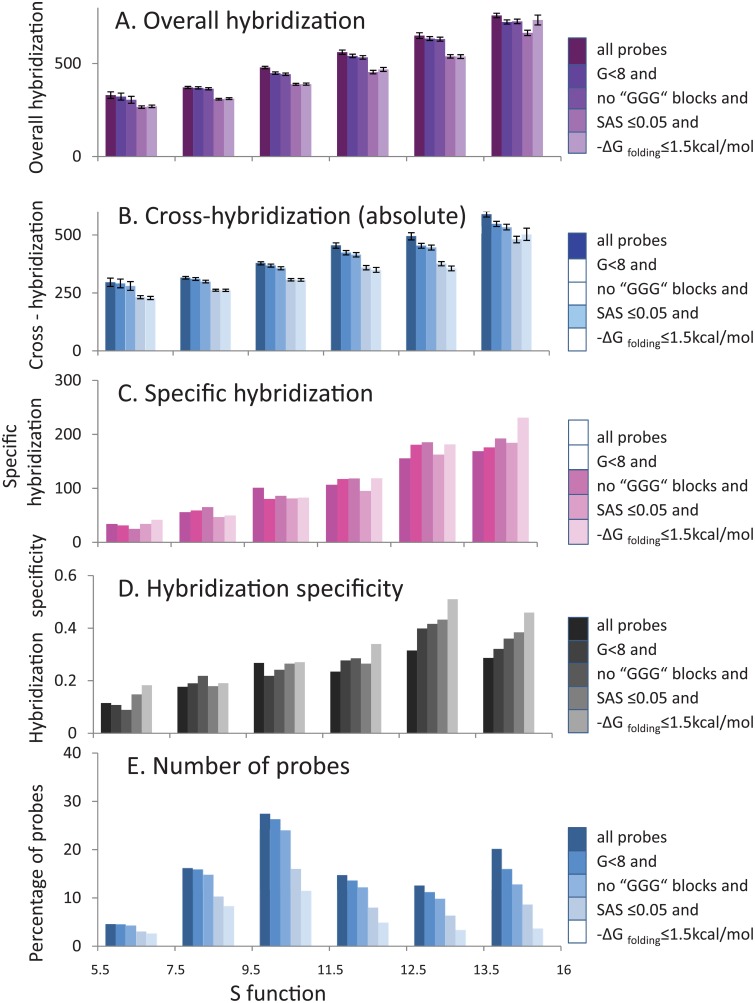
Categorization of probes by theoretically estimated *S* function after filtration using all analyzed parameters. Oligoprobes were categorized into bins according to the estimated *S* function, where *S* is calculated as a combination of genomic occurrence of all *k*-mers (7 ≤ *k* ≤ 11) in an oligoprobe and their binding energy (see Material and methods for details). Averaged hybridization signals and other hybridization characteristics calculated for each bin are shown as columns of assorted colors depending on the bin type. The columns with variable shades of a particular color illustrate the filtration process that gradually removes from the database all probes with a defined sequence characteristic, which negatively affect hybridization specificity because of involvement in parallel hybridization reactions. Colors of the columns become lighter after each filtration step. The darkest columns represent all probes from the database, while lightest columns represent probes after all filtration steps. The filtration process entails the following sequential probe removal steps: 8 or more of G in a sequence, at least one GGG block, SAS score above 0.05, -*ΔG* folding above 1.5 kcal/mol. A. Overall hybridization (derived from the probe’s dataset with specific targets). B. Cross-hybridization (absolute) (derived from the dataset of probes without specific targets). C. Specific hybridization (represented by subtraction values between overall and cross-hybridizations). D. Hybridization specificity (represented by ratio between specific and cross-hybridization). E. The percentage of probes (from complete dataset) in each bin.

We grouped oligoprobes according to the estimated function *S* (Fig 7), which can vary from 5 to 16. We showed that increases in bin ranges for the function *S* are simultaneously accompanied by an increase in averaged values of both overall hybridization and absolute cross-hybridization signals (Fig 7A and 7B). Probes’ specific hybridization (Fig 7C) and hybridization specificity, which is a ratio of specific- versus cross- hybridization (Fig 7D), also demonstrated a growing trend. However, the hybridization specific signal increases significantly faster in comparison to the cross-hybridization (Fig 7A and 7B). The exclusion of probes with negative characteristics from the analysis resulted in the pronounced mutual dependence between hybridization specificity and the function *S* (Fig 7D).

### Inter-relationships between probes hybridization and sequence characteristics

#### Absolute and relative cross-hybridization

The study of trends of absolute and relative cross-hybridization values shows that considering both parameters at once can result in conflicting trend outcomes. Thus, the study highlights the need to differentiate the two terms. Analysis of the binned averaged signals of probes revealed that both absolute and relative cross-hybridizations have growing trends along with G-count or an increase in self-folding vulnerability (Figure A-C in S2 Fig). Both showed a growing trend with an increase in SAS score, so smaller nucleotide bias corresponds to smaller absolute and relative cross-hybridization (Figure B in S2 Fig). In contrast, both variables showed opposite trends with an increase in probes’ binding energy; absolute cross-hybridization has a growing trend, whereas relative has a decreasing trend (Figure D in S2 Fig).

#### Hybridization specificity and absolute cross-hybridization

Both hybridization characteristics might change according to either similar or different trends, depending on the probes’ categorization. Hybridization specificity has a descending trend, while absolute cross-hybridization has a growing trend, along with the probes’ categorization according to the G-count or self-folding vulnerability increase (Figure A-C in S3 Fig). Absolute cross-hybridization has a growing trend, while hybridization specificity has a decreasing trend along with an increase of the SAS score in bins (Figure B in S3 Fig). Consequently, hybridization specificity and absolute cross-hybridization trends of changes are opposite to each other in all the relationships mentioned above. In contrast, they are similar along with an increase of probes’ binding energy; and both hybridization specificity and absolute cross-hybridization demonstrate growing trends. However, these trends’ slopes are significantly different, since specific signal increases faster in comparison with cross-hybridization (Figure D in S3 Fig).

#### Summary of all analyzed inter-relationships between probes sequence and hybridization characteristics

Analysis of all trends presented above demonstrated that absolute and relative cross-hybridization changing trends might be similar or opposite of each other depending on the sequence characteristic change. The same is true for hybridization specificity and absolute cross-hybridization. However, even when the trends are similar in directions, their magnitudes may be different. Such differences characterize the relationships between probes’ hybridization characteristics and their binding energy. The directions of all trends are summarized in Table 1. Detailed analysis of changes in absolute and relative cross-hybridization trends according to different sequence characteristics is promising for optimization of oligoprobe design.

**Table 1:** Relationships between hybridization values and probes’ sequence characteristics.

Name of a probe sequence feature	Overall-combined hybridization	Absolute	Relative	Hybridization specificity
		cross-hybridization		
G-count (above 3n) ↑	↑	↑	↑	↓
G-block presence ↑	↑	↑	↑	↓
Positions 1–4 from 5 ' end ↑	↑	↑	↑	↓
Positions 5–23 from 5' end ↑	↑	no change	↑	↓
SAS score ↑	↑	↑	↑	↓
Self-folding vulnerability ↑	no change	↑	↑	↓
Binding energy ↑	↑	↑	↓	↑
Genomic occurrence of *k*-mers in a probe ↑	↑	↑	↑	↓

### Algorithm for selection of specific probes

Less specific probes, which are insensitive to gene copy number variations, are those that participate very little in any binding reactions and those that actively participate in off-target hybridization reactions. These probes have one or more negative characteristics: low binding energy, high self-folding vulnerability, high G-content, presence of GGG-blocks, higher SAS score and high values of 11-mers occurrence in a human genome. We suggest an algorithm for elimination of such probes from a set of design candidates. The cutoff points for input parameters could be user defined. We suggest eliminating probes with the following characteristics (default parameters): (i) -*ΔG*_binding_ below 21 kcal/mol or -*ΔG*_folding_ above 3 kcal/mol; (ii) SAS score above 0.05; (iii) G-count above 7 nt; (iv) occurrence of two GGG-blocks anywhere in a probe sequence; (v) one GGG block being present at any of the first four nucleotide positions from probe’s 5’ end; (vi) genomic occurrence of 11-mers above 250 (here and below, minimum value of genomic occurrence among all 11-mers in a probe was applied, see Materials and methods).

Each negative characteristic with the indicated cut-off point diminishes hybridization specificity by a factor of at least two. Thus, the hybridization specificity of remaining probes is two times higher in comparison with the hybridization specificity of filtered probes (0.25 versus 0.12). Approximately 50% of probes analyzed in this work have at least one of these negative sequence characteristics. The “specific probes” or “optimal probes” are those that do not have any negative characteristics, or by definition, these probes contain the following characteristics:

-*ΔG*_binding_ above 23.5 kcal/mol;
- *ΔG*_folding_ below 1.5 kcal/mol;
3. SAS score below 0.05;
4. G-count below 8 nt;
5. no GGG-blocks.
6. genomic occurrence of 11-mers below 250.

The hybridization specificity of these selected probes is approximately two times higher in comparison with the hybridization specificity of remaining probes (0.5 versus 0.25). Approximately 4% of probes considered in this work belong to the “specific” category (hybridization specificity ~0.5).

There is an option to classify the oligoprobes based on the estimated values of *S* (Fig 7), instead of using -*ΔG*_binding_ and 11-mers occurrence. The analysis of optimal bins with higher values of *S* (threshold– 12.5) allows the user to extract a larger number of specific probes (8%). The default parameters for the specific probe design are following:

1. *S* above 12.5;
- *ΔG*_folding_ below 1.5 kcal/mol;
3. SAS score below 0.05;
4. G-count below 8 nt;
5. no GGG-blocks.

## Discussion

In this study, we demonstrated that elevated levels of hybridization specificity and absolute cross-hybridization are not mutually exclusive and may be attributed to the same probe sets. Moreover, trends in which hybridization specificity and absolute cross-hybridization are changing along with a sequence characteristic can differ significantly. They might be either both positive, either both negative, or opposite of each other, depending on the sequence probe characteristic.

Low cross-hybridization is not necessarily indicative of a probe’s high specificity. The low value of cross-hybridization is frequently a result of the poor ability of the probe to interact in general; in such cases, both specific and cross-hybridizations are low. Conversely, the probes that generate high specific hybridization could also generate high cross-hybridization because of their better interaction ability, both on- and off-target. It is important to differentiate between absolute and relative cross-hybridization values that also account for the specific hybridization. Some published studies suggest avoiding probes with high binding energy because of their high vulnerability to cross-hybridization [27]. Our study disproves this concept; probes with high binding energy may have high specific as well as high absolute cross-hybridization (at least for the Affymetrix platform). The ratio between specific and cross-hybridization signals is high for such probes. Thus, prediction of relative cross-hybridization is more important than prediction of absolute cross-hybridization for specific probe selection.

Specific probe-target interactions occur in parallel with non-specific interactions of two types, WC and non-WC. Potential off-target interactions of probes based on WC pairing are mainly evaluated through estimation of target *k*-mers in human genomes, binding energy, and partially through SAS scores and self-folding evaluation of probes, while their off-target interactions based on non-WC pairing are partially evaluated through assessment of G-effects. These evaluations are helpful for detection of probes that are involved in off-target hybridization reactions and for removal of such probes from the pool of all probes. Such filtering significantly improves hybridization specificity of the remaining probes on average, and could be used during the array design process. The improvement is more pronounced in the category of the probes with high binding energy because of the proportion of the probes involved with parallel hybridization reactions is larger in this category compared to ones with lower binding energy. These probes have high nucleotide bias: they are GC-rich and capable of stable self-folding, specifically, enriched with G and GGG blocks.

This study demonstrates that bins of probes with high binding energy are enriched with more specific oligos and show significantly greater averaged hybridization specificity. However, an increase of target concentration (gene copy numbers for CGH) may also affect the behavior of the probes with high binding energy, where the trend may be different, or hybridization specificity may reach a plateau. Hadiwikarta and co-authors [46] stated before that “For a given set of experimental parameters the affinity window of probe—target interaction is always limited … changes in experimental conditions can easily bring some measurements out of detection range.” High probe’s binding energy, which is optimal for evaluation of low target concentration, might be sub-optimal for high target concentrations. Moreover, if a target concentration is too high, other artifacts are possible; probes with high binding energy might achieve hybridization saturation or generate signals that are higher than a scanner’s upper detection limit. Thus, array probes should be designed in a certain range of binding energies for measuring the widest range of target concentrations. Consequently, isothermal array probes (the probes that share the same melting temperatures and binding energies) [47] are not optimal for measuring target concentrations because of narrow measuring range.

Many bio-techniques, beyond microarrays technology, rely on specific interactions of the oligoprobe with complementary targets. Even though the results of the study presented here are derived from microarray experiments, the physicochemical principals underlying our findings are “microarray-free” and most of the steps in the design procedure may be extrapolated universally.

## Conclusions

Hybridization specificity and absolute cross-hybridization values of oligoprobes both increase with increasing binding energy of probes in the analyzed bins. We showed that high specific hybridization and high cross-hybridization are not mutually exclusive, and may be attributed to the same probe sets. In other words, the level of non-specific interactions for some molecules may be high, but the ratio between off-target and total hybridization signals may be low. This also means that the specific signal is sufficient in magnitude for a high on-target/off-target ratio, which defines interaction specificity.

Low hybridization specificity of a probe is related to its high self-folding vulnerability, nucleotide composition bias, G-richness and GGG-block presence. Probes with these characteristics have high absolute and high relative cross-hybridization values. High genomic occurrence of *k*-mers in an oligoprobe decreases the probe’s hybridization specificity.

In this study, we suggested applying the function *S* as the combination of probe binding energy and occurrence of probe’s *k*-mers (7 ≤ *k* ≤ 11) in the genome for efficient oligoprobe design. Along with an increase of *S* function of probes, both averaged hybridization signals (specific and cross-hybridization) exhibit growing trends, where the hybridization specific signal increases significantly faster in comparison to the cross-hybridization. The application of *S* allows extracting a larger number of specific probes, as compared to using only binding energy. Thus, the *S* function together with other described sequence characteristics are promising features for further improvement of oligoprobe design for bio-techniques that require selection of most specific probes.

## Supporting information

S1 Fig Relationships between relative cross-hybridization values and probes’ sequence characteristics. Oligoprobes were categorized into bins according to their variable sequence characteristics. The relative cross-hybridization value was calculated for each bin as an average ratio of absolute cross-hybridization versus overall-combined hybridization value. Relationship between probes’ relative cross-hybridization values and A) the numbers of G in the probes; B) the numbers of GGG-blocks in the1^st^ positions of the probes; C) the numbers of GGG-blocks located in 2^nd^ to 4^th^ positions of the probes; D) the numbers of GGG-blocks located in in any position from the 5^th^ to 23^rd^; E) probes’ SAS score; F) self-folding vulnerabilities in the probes; G) probes’ binding energies.
(EPS)

S2 Fig Absolute and relative cross-hybridizations versus probes’ sequence characteristics. Oligoprobes were categorized into bins according to variable probes characteristics. Averaged absolute or relative cross-hybridization signals were calculated for each bin and their values are shown as columns. Absolute cross-hybridization values are shown on the primary Y axis and relative cross-hybridization is shown on the secondary Y axis. Categorization according to A) G-count (all probes); B) SAS score (all probes); C) self-folding vulnerability (all probes); D) probes categorization according to binding energy. Probes with characteristics that negatively affect their hybridization specificity were excluded from the categorization procedure. The exclusion criteria were -*ΔG*_folding_ above 1.5 kcal/mol, SAS score above 0.05, or G-count above 7 nucleotides, and GGG-block(s) presence.
(EPS)

S3 Fig Absolute cross-hybridization and hybridization specificity versus probes’ sequence characteristics. Oligoprobes were categorized into bins according to variable probes’ sequence characteristics. Averaged absolute cross-hybridization signals or hybridization specificity were calculated for each bin. Absolute cross-hybridization values are shown according to primary Y axis and hybridization specificity is shown on the secondary Y axis. All probes were categorized according to A) G-count; B) SAS score; C) probes’ self-folding vulnerability; D) probes’ binding energy. Probes with characteristics that negatively affect their hybridization specificity were excluded from the categorization procedure. The exclusion criteria were the same as described in S2 Fig.
(EPS)
